# Validation of dynamic three-dimensional whole heart magnetic resonance myocardial perfusion imaging against single photon emission computed tomography for the detection of functionally significant coronary heart disease

**DOI:** 10.1186/1532-429X-14-S1-O47

**Published:** 2012-02-01

**Authors:** Roy Jogiya, Geraint Morton, Yasmine Samaroo, James Otton, Eike Nagel, Sebastian Kozerke, Stephen R Underwood, Sven Plein

**Affiliations:** 1 Kings College London, London, UK; 2 ETH, Zurich, Switzerland; 3 LIGHT Institute, Leeds, UK; 4 Imperial College, London, UK

## Summary

We demonstrate the feasibility of 3D myocardial perfusion CMR at 3 Tesla against single photon emission computer tomography for the detection and estimate of ischaemic burden and show good agreement between the techniques. This novel technique shows promising use of this method in a small cohort of patients to estimate ischaemic burden for purpose of risk stratification of patients with known or suspected coronary disease.

## Background

The extent and severity of ischaemia on single photon computed tomography (SPECT) is commonly used to risk-stratify patients with suspected coronary artery disease (CAD). Accurate estimates of ischaemia burden by CMR is limited because conventional two-dimensional myocardial perfusion methods cover the heart in a limited number of non-contiguous sections. More recently, three-dimensional (3D) myocardial perfusion CMR has been proposed to overcome the limitation of spatial coverage but has yet to be validated against SPECT.

## Aims

To compare ischaemia burden on 3D myocardial perfusion CMR with (99m)Tc-tetrofosmin myocardial perfusion SPECT (MPS).

## Methods

Ten consecutive patients with known or suspected CAD who were clinically referred for MPS underwent 3D CMR perfusion. The 3D datasets were analysed by an experienced observer blinded to the MPS data and images were scored for the presence of inducible ischaemia in accordance to the AHA 17 segment model.

Semi-quantitative analysis of the ischaemic burden was calculated from the sum stress difference between stress and rest tracer uptake for MPS. The 3D CMR perfusion images were similarly analysed for inducible perfusion abnormalities (all areas of hypoperfusion excluding scar on LGE).

## Results

**Figure 1:**
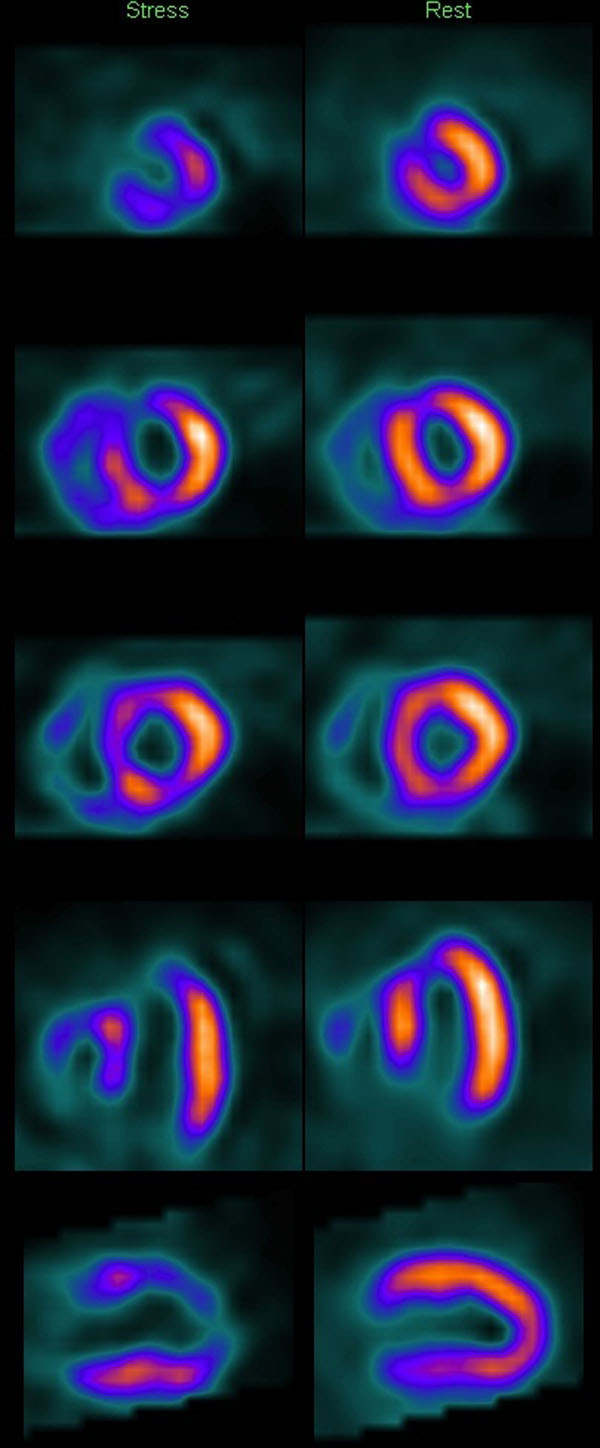
SPECT image demonstrating reduced tracer uptake in the anterior and inferior walls at stress. Note extension into the apex which is also apparent with whole heart 3D CMR perfusion.

**Figure 2:**
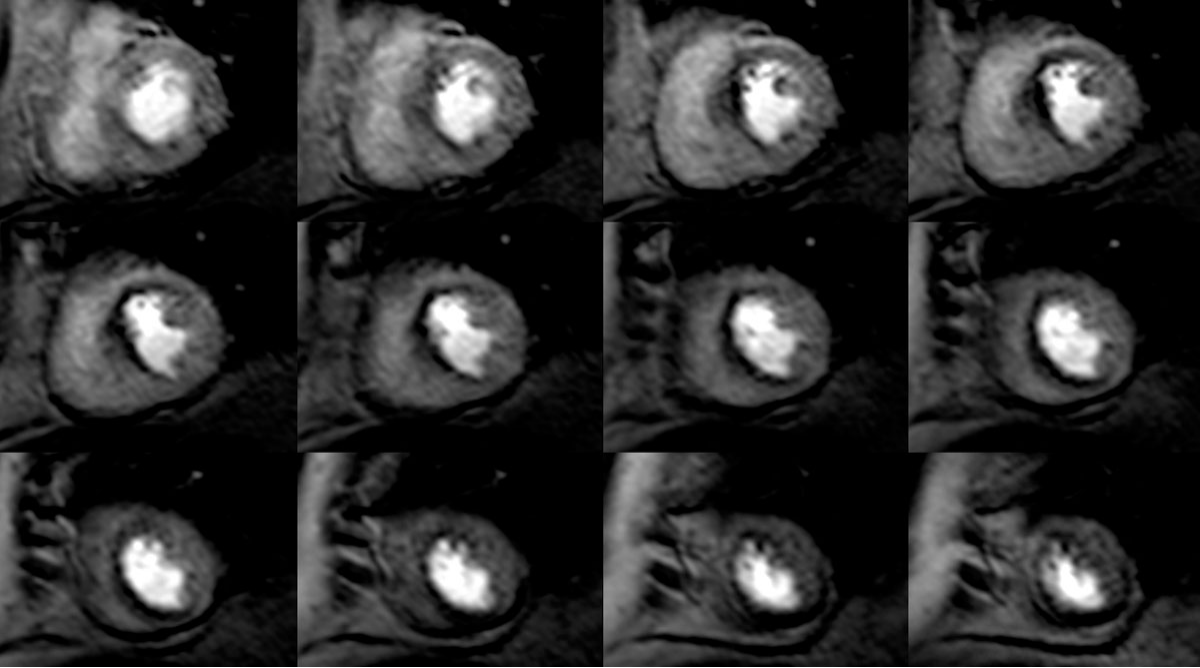
3D CMR perfusion of the same patient demonstrating good anatomical agreement of the perfusion defect and ischaemic burden by semi-quantitative scoring.

3D myocardial perfusion CMR and MPS agreed in nine of the ten patients for the detection of any inducible ischaemia. In one patient, CMR detected ischaemia not seen on MPS. Preliminary diagnostic sensitivity was 100%, specificity 75%, postive predictive value 0.86, negative predictive value 1.0) using SPECT as the gold standard. The mean percentage of inducible ischaemia was 7.3% (range 0-22.2%) for CMR and 10.3% for SPECT (range 0-25.0%). Overall there was no significant difference (P=0.47)(Figures 1 and 2).

## Conclusions

3D myocardial perfusion CMR agrees well with SPECT for the detection of coronary artery disease. Both techniques produced similar volumes of ischaemia in the small cohort studied. 3D myocardial perfusion CMR offers a promising alternative method of detecting ischaemia with the added benefits of improved spatial resolution and avoiding the need for ionising radiation. The method holds promise for the risk stratification of patients with known or suspected CAD.

## Funding

SP is funded by British Heart Foundation fellowship FS/10/62/28409.

SP/EN receives research grant support from Philips Healthcare.

